# Perceptions of C-reactive Protein Measurement Among General Physicians: A Qualitative Study on Diagnostic Value, Clinical Dilemmas, and Professional Growth

**DOI:** 10.7759/cureus.63695

**Published:** 2024-07-02

**Authors:** Ryuichi Ohta, Toshihiro Yakabe, Chiaki Sano

**Affiliations:** 1 Community Care, Unnan City Hospital, Unnan, JPN; 2 Family Medicine, Unnan City Hospital, Unnan, JPN; 3 Community Medicine Management, Shimane University Faculty of Medicine, Izumo, JPN

**Keywords:** rural, diagnostic techniques and procedures, practice, attitudes, health knowledge, clinical decision-making qualitative research, primary care, general physicians, c-reactive protein

## Abstract

Introduction

C-reactive protein (CRP) is a widely used laboratory test for assessing infections, inflammatory diseases, and malignancies, playing a critical role in clinical diagnosis and management. Despite its utility, CRP measurement practices vary among physicians, often influenced by training and clinical experience. This study explores general physicians' perceptions of CRP measurement in clinical practice, focusing on its diagnostic value, associated dilemmas, and impact on clinical growth and decision-making.

Methods

This qualitative study employed thematic analysis to examine the perceptions of general physicians at Unnan City Hospital, Unnan, Japan regarding CRP measurement. Sixteen general physicians were selected through purposive sampling and participated in one-on-one semi-structured interviews. The interviews were conducted in Japanese, recorded, transcribed verbatim, and analyzed inductively to identify themes. The analysis involved iterative coding and extensive discussion among the research team to ensure the reliability and validity of the findings.

Results

Three main themes emerged from the analysis: the usefulness of CRP for diagnosis and collaboration, dilemmas associated with CRP usage, and clinical growth through reconsideration of CRP's importance. Physicians highlighted CRP's value in distinguishing inflammatory from non-inflammatory diseases, predicting clinical courses, and facilitating communication with specialists. However, dilemmas arose from discrepancies between CRP levels and clinical symptoms, the influence of various non-specific factors, and habitual testing driven by training, leading to unnecessary tests and diminished clinical skills. Participants recognized the need to view CRP as one of many diagnostic tools, cultivate a habit of questioning its necessity, and reflect on its use to enhance clinical reasoning and professional growth.

Conclusions

CRP measurement is a valuable diagnostic tool, but effective use requires a balanced and critical approach. Discrepancies between CRP levels and clinical symptoms can lead to over-reliance on laboratory results and unnecessary testing. General physicians should integrate CRP within a broader diagnostic framework, combining it with patient history, physical examination, and other tests. Reflecting on the necessity and implications of CRP measurements can improve clinical reasoning and decision-making, ultimately enhancing patient care and resource management. Future research should explore similar perceptions in diverse healthcare settings and develop strategies to optimize CRP use in clinical practice.

## Introduction

C-reactive protein (CRP) is one of the most utilized laboratory tests for assessing various patient conditions, including infections, inflammatory diseases, and malignancies [1]. Its widespread use in clinical settings underscores its importance as a diagnostic tool [2]. Previous studies have demonstrated that CRP can be instrumental in diagnosing various diseases and ruling out critical conditions [3,4]. The correlation between CRP levels and the severity of infectious and inflammatory diseases is well-established, providing valuable information for clinicians in managing patient care [3,4].

Physicians often measure CRP as a routine practice, recognizing its significance in the clinical dialogue among medical professionals [5]. Measuring CRP levels can facilitate discussions about patient management strategies, aiding decision-making [6]. However, it is crucial to acknowledge that CRP levels can vary significantly depending on individual patient characteristics and the clinical course of each disease [7,8]. This variability can sometimes lead to misleading interpretations if not considered carefully within the context of the patient's overall clinical picture.

General physicians, who are system-specific specialists, play a pivotal role in advocating for the judicious use of medical resources, including laboratory markers like CRP. The "Choosing Wisely" campaign, which promotes the rational use of medical tests and procedures, underscores the need for prudent CRP measurement [9]. Despite the general awareness of CRP's diagnostic value, its measurement practices can vary widely among physicians. They are often used in diverse ways or as a routine part of clinical assessment, which should be intervened comprehensively [10].

General physicians can critically use CRP measurements to optimize diagnosis and treatment. Understanding CRP from multiple perspectives can improve the prediction of clinical courses, including its pathophysiological implications, the nuances of its variability, and its role in different clinical scenarios [4,11]. The usage of CRP measurement can vary because clinical perceptions regarding CRP measurement are typically shaped by physicians' clinical experiences and educational backgrounds.

The primary aim of this study is to elucidate general physicians' perceptions and the change of CRP measurement in assessing patient conditions through clinical experiences in general medicine. By exploring these perceptions, the study seeks to identify how general physicians integrate CRP testing into their diagnostic processes and how their clinical experiences and education influence their use of CRP. Understanding these factors and changes in the perceptions can help develop strategies to enhance CRP's practical use, ensuring that it contributes meaningfully to patient care and resource management. This research aims to contribute to the broader discourse on the rational use of laboratory tests and to support general physicians in making informed decisions that benefit patient outcomes.

## Materials and methods

This research utilized thematic analysis anchored in relativist ontology and constructivist epistemology [12]. The study aimed to understand how physicians' diverse backgrounds, experiences, and education influence their perceptions of measuring CRP. Specifically, it examined the variations in general physicians' views and the factors affecting these perceptions, all within a qualitative research methodology [13].

Setting

In 2022, Unnan City had a population of 35,738, with 40.27% of residents aged 65 or older. The city's main healthcare provider, Unnan City Hospital, had 281 beds distributed across acute, general, rehabilitation, and chronic care units. The Department of Family Medicine collectively oversees patients requiring internal medicine [14].

Participants

The study involved general physicians who had experience working at Unnan City Hospital. Participants were selected using purposive sampling. Inclusion criteria required experience in general medicine, willingness to participate, and ability to engage in interviews, while exclusion criteria included lack of consent, experience in general medicine, and inability to participate in interviews. Informed consent was obtained from all participants. The study ultimately included 16 general physicians.

Data collection

We conducted one-on-one semi-structured interviews with general physicians at Unnan City Hospital, all conducted in Japanese. Each interview lasted approximately 60 minutes and was facilitated by the first researcher (RO), who has expertise in family medicine, medical education, and public health. Before beginning the semi-structured interviews, RO established rapport with the participants. The interview guide included questions about the recent usage of CRP, its advantages and disadvantages, challenges in decision-making involving CRP, factors influencing the measurement of CRP, and any changes in perceptions regarding its use. Each interview was recorded and transcribed verbatim.

Analysis

The study employed an in-depth inductive thematic analysis to investigate themes related to general physicians' perceptions of measuring CRP in general medicine contexts [13,15]. Interviews were meticulously recorded and transcribed verbatim, followed by a comprehensive analysis. After every three interviews, RO and TY independently read the transcripts to code and identify emerging patterns and themes. This iterative coding process deepened the understanding and refinement of the data with each review. RO initially created a preliminary codebook, continually adapting based on evolving insights from the transcripts. Concurrently, TY independently reviewed the transcripts and contributed to the coding process. The researchers engaged in extensive discussions to compare and contrast their findings, merge codes, and refine themes, ensuring diverse perspectives in data interpretation. The coding and discussion process continued until saturation was reached, with no new themes emerging. At this final stage, CS, a specialist in community care, joined the analysis, adding another level of expertise and validating the themes for their applicability in community care contexts. After finalizing the themes, the results were translated from Japanese to English. This translation was meticulously undertaken to preserve the nuances and context of the original data, ensuring accurate communication of the findings to an international audience.

Reflexivity

The diverse expertise within the research team significantly enriched the research process, promoting collaborative interactions between researchers and participants.

The team included RO, a family physician and public health professional with a master’s degree in public health and family medicine; TY, the director of a non-profit organization with extensive experience in rural community support; and CS, a medical educator specializing in community healthcare management. Each member brought unique insights: RO provided practical experience in rural community health, TY offered a grassroots perspective from decades of supporting isolated individuals in communities, and CS contributed an academic and systematic approach to community healthcare management and education.

The team engaged in rigorous discussions to ensure an unbiased approach, continually challenging and refining each other's ideas. This process involved exploring alternative viewpoints and reflecting on how their professional and personal experiences might influence data analysis. This reflective practice was critical in mitigating potential biases, fostering a more objective and comprehensive understanding of the study's findings.

The team's engagement with participants was marked by respect and empathy, valuing shared insights and experiences. This participatory approach facilitated a deeper understanding of the nuances in rural healthcare perceptions and practices, enhancing the study's overall depth and relevance.

Trustworthiness

Credibility was established through prolonged engagement with participants and iterative discussions. Regular peer debriefing among the research team ensured cross-validation of findings. Detailed context descriptions, participant demographics, and methodology were provided to support transferability to other rural settings. An audit trail was maintained, documenting the research process from data collection to analysis, thereby minimizing researcher bias. The study's dependability was reinforced by a consistent and transparent methodological approach, with a reflexive critique by the research team enhancing reliability.

Ethical consideration

The Unnan City Hospital Clinical Ethics Committee approved the study protocol (20240003).

## Results

The thematic analysis identified three main themes regarding general physicians' perceptions of CRP measurement in general medicine: usefulness for diagnosis and collaboration, dilemmas associated with CRP usage, and clinical growth through reconsideration of CRP's importance. Firstly, CRP was highlighted as a valuable tool for distinguishing between inflammatory and non-inflammatory diseases, predicting clinical course, and facilitating communication with specialists. Secondly, physicians experienced dilemmas due to discrepancies between CRP levels and clinical symptoms, the influence of various factors on CRP, habitual testing driven by training, and the resulting increase in unnecessary tests and potential decline in clinical skills. Lastly, physicians acknowledged the need for clinical growth by treating CRP as one of many diagnostic tools, questioning its necessity, and refining their clinical reasoning skills through critical evaluation of its use (Table 1).

**Table 1:** The result of the thematic analysis regarding the perception and changes of general physicians regarding measuring CRP in general medicine contexts. CRP: C-reactive protein

Themes	Concepts
The Usefulness for Diagnosis and Collaboration	Distinguishing Inflammatory From Non-inflammatory Disease
	Facilitating Communication With Specialists
	Predicting Clinical Course
Dilemma for the Usage of CRP	Conflict Arising From Discrepancies With Clinical Symptoms
	Impact of Various Influencing Factors
	Habitual Use Driven by Training and Expectations
	Increase in Unnecessary Testing
	Diminished Clinical Skills
Clinical Growth Through Reconsideration of CRP’s Importance	Viewing CRP as One of Many Diagnostic Tools
	Cultivating a Habit of Questioning Necessity
	Refining Clinical Reasoning

**Figure 1:**
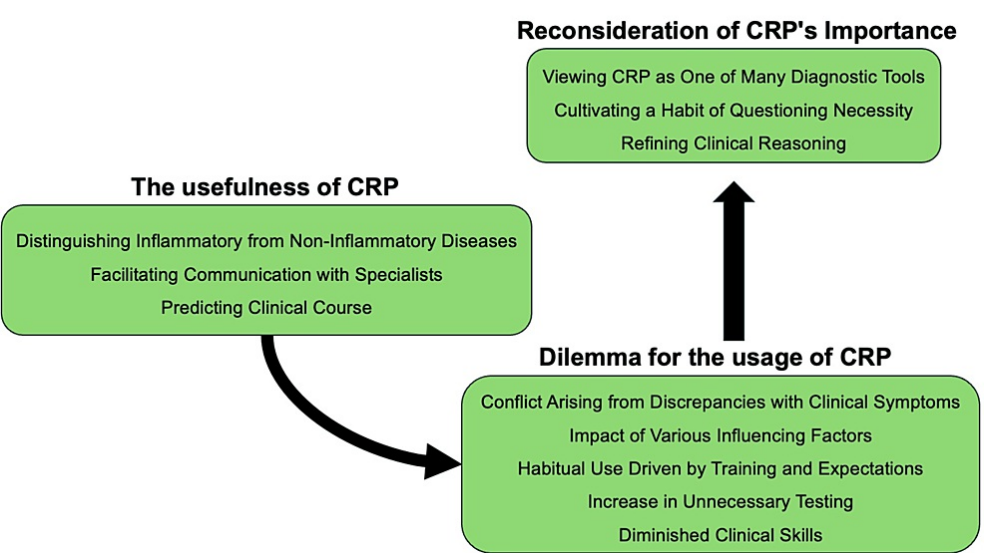
Conceptual figure of the changes in the perception of general physicians regarding measuring CRP in general medicine contexts CRP: C-reactive protein; Image Credits: Ryuichi Ohta

Figure 1 shows a conceptual figure of the changes in the perception of general physicians regarding measuring CRP in general medicine contexts.

Usefulness for diagnosis and collaboration

Distinguishing Inflammatory From Non-inflammatory Diseases

Participants emphasized the effectiveness of CRP in differentiating between inflammatory and non-inflammatory diseases. One participant remarked, "When a patient comes in with vague symptoms, CRP helps us quickly narrow down the possibilities. It's often our first go-to test" (Participant 7). This sentiment was echoed by another physician who explained, "CRP provides a clear starting point. If the levels are high, we know to look more closely for an underlying inflammatory condition. If they're normal, we can consider other non-inflammatory causes" (Participant 2). Another participant added, "In cases where symptoms are not specific, having a CRP result can be a game-changer. It reduces the guesswork and directs our diagnostic approach more efficiently" (Participant 4). These insights underscore CRP's pivotal role in the initial assessment and decision-making process, particularly when faced with nonspecific clinical presentations in general practice. For the participants, measuring CRP could be the starting point for diagnosing patients with vague symptoms without focusing on investigations.

Facilitating Communication With Specialists

CRP was recognized as a universal marker well-understood by various specialists, which aids in conveying the severity and urgency of a patient's condition. One physician explained, "When I show a specialist the CRP results, they immediately grasp the severity. It simplifies the whole referral process" (Participant 2). Another participant added, "CRP is like a common language we all speak. Whether it's a cardiologist or an infectious disease expert, they all understand the implications of a high CRP level" (Participant 5). This shared understanding was crucial in ensuring timely and effective patient care. "Having a high CRP result on hand helps me convey the urgency of the situation without lengthy explanations," (Participant 1) noted a physician. Another highlighted, "Although some CRP measurements may not be needed to assess patients’ conditions, it speeds up communication. Specialists know what to expect and can prepare accordingly, which is particularly important in acute or critical cases" (Participant 3). These quotes illustrate how CRP measurements facilitate smoother and more efficient communication among healthcare providers, ultimately enhancing patient management and care coordination. General physicians know that some cases may not need the measurements of CRP to assess the medical conditions, but measurement was used for effective communications with other physicians.

Predicting Clinical Course

General physicians frequently use CRP to monitor disease progression or improvement. An increase in CRP levels often signaled worsening conditions, while a decrease provided reassurance. One participant reflected, "Seeing a drop in CRP gives us a sense of relief. It means our treatment is working, and the patient is improving" (Participant 4). Another physician elaborated, "CRP trends are incredibly informative. When the levels rise, it's a red flag that we might need to adjust our treatment strategy. Conversely, a decline in CRP is a positive indicator that reinforces our current approach" (Participant 7). Another participant shared, "Monitoring CRP levels over time helps us track the patient's response to treatment. It acts as a gauge for the effectiveness of our interventions and informs our next steps" (Participant 9). These insights highlight CRP's vital role in guiding clinical decisions and providing a measurable indicator of patient outcomes during treatment. Frequent usage of CRP made general physicians dependent on the measurement and value of assessing patients' conditions, and they faced various dilemmas in assessing patients’ conditions effectively.

Dilemma for the usage of CRP

Conflict Arising From Discrepancies With Clinical Symptoms

Physicians experienced confusion when CRP results did not align with the patient's clinical presentation. Instances where CRP levels remained high despite visible patient improvement caused particular frustration. One physician mentioned, "It's perplexing when CRP is elevated, but the patient looks and feels better. It makes me question my reliance on the test" (Participant 3). Another physician added, "There are times when the patient is clinically improving, yet the CRP levels don't reflect that progress. It can be quite confusing and sometimes leads us down the wrong diagnostic path" (Participant 3). A participant also shared, "I've had cases where the CRP remained high for reasons we couldn't immediately identify, which added unnecessary complexity to patient management" (Participant 3). These quotes underscore physicians' challenges when CRP results contradict clinical observations, leading to potential uncertainty and over-reliance on lab results over clinical judgment. Although CRP can be a helpful test in simple clinical situations, complicated cases in general medicine show various changes in the value, which confuses general physicians when assessing clinical courses.

Impact of Various Influencing Factors

Participants understood that multiple factors could cause CRP elevations, often unrelated to the patient's illness. This led to anxiety and unnecessary investigations. "There are times when CRP rises for reasons we can't pinpoint immediately, leading to more tests and stress for both the patient and me," (Participant 10) admitted a participant. Another physician reflected, "Sometimes, CRP levels increase due to factors like minor infections or inflammation that aren't clinically significant, but we feel compelled to investigate further, just to be sure" (Participant 4). A participant added, "We know that various non-specific factors can influence CRP, but it still creates a sense of unease and prompts additional testing, which might not always be necessary" (Participant 2). These observations highlight the complexity of interpreting CRP levels and the resulting impact on clinical decision-making, often leading to a cascade of further tests and increased patient anxiety. The participants considered that inappropriate measurement of CRP confused their judgment and that assessment of CRP should be adjusted to each patient’s clinical conditions to reduce needless investigation.

Habitual Use Driven by Training and Expectations

Many physicians attributed their frequent CRP ordering to habits formed during their initial training and expectations from senior doctors. "Even if I think a CRP test isn't needed, I order it out of habit and fear of reprimand from my supervisors" (Participant 1), shared a participant. Another physician reflected, "During my early years of training, we were taught to rely heavily on CRP. It became a default part of our diagnostic process" (Participant 6). This ingrained practice was reinforced by the clinical culture and expectations from more experienced colleagues. "There were times when not ordering a CRP test felt like a deviation from the norm, almost like I was neglecting a part of patient care" (Participant 8), noted another participant. A further comment highlighted the internal conflict, "I often find myself ordering CRP reflexively, even when I know it's not clinically necessary, simply because it's what we've always done" (Participant 1). The participants realized that their educational backgrounds affected their perception of CRP measurements, misleading their assessment of patients’ clinical conditions and excessive testing. These insights reveal how training and professional expectations contribute to the habitual use of CRP, sometimes leading to over-testing and reinforcing reliance on the marker beyond its necessary scope.

Increase in Unnecessary Testing

The reliance on CRP often led to additional tests, even when the patient's condition seemed stable. This over-testing was acknowledged as a source of wasted resources. "When CRP doesn't match my clinical judgment, I end up ordering more tests than necessary, which isn't ideal," (Participant 2) reflected a physician. Another participant explained, "Sometimes, an unexpected rise in CRP makes me second-guess my initial assessment, prompting a slew of additional tests to rule out potential issues" (Participant 5). This practice not only strained resources but also increased patient anxiety and inconvenience. "I know that ordering more tests because of an unexpected CRP result can lead to unnecessary procedures and costs, but it's difficult to avoid when there's an underlying uncertainty," (Participant 3) noted another doctor. The variety of changes in CRP confuses general physicians, triggers needless tests, and increases the complexity of considering patients’ conditions. These reflections highlight the unintended consequences of over-relying on CRP, emphasizing the need for more balanced and judicious use of this diagnostic tool to prevent unnecessary testing and resource wastage.

Diminished Clinical Skills

Dependence on CRP measurements was perceived to erode physicians' clinical skills, as they might prioritize lab results over direct patient observations. "Relying too much on CRP can make us lazy. We need to remember the importance of physical exams and patient history," (Participant 9) emphasized a participant. Another physician shared, "I've noticed that my clinical acumen dulls when I over-rely on CRP. It’s essential to balance lab results with a thorough examination and patient interaction" (Participant 7). This sentiment was echoed by another participant who remarked, "The convenience of CRP testing can sometimes overshadow the critical thinking and hands-on skills that are fundamental to good clinical practice" (Participant 1). Dependence on the result of CRP made general physicians’ mental distance from bedside examinations, weakening clinical skills. These insights suggest that while CRP is a valuable tool, its overuse can detract from essential clinical skills, underscoring the importance of integrating lab results with comprehensive patient evaluation to maintain high standards of care.

Clinical growth through reconsideration of CRP's importance

Viewing CRP as One of Many Diagnostic Tools

Participants recognized the need to treat CRP as a single component within a broader diagnostic framework, fostering a more holistic approach to patient care. "CRP should be one piece of the puzzle, not the whole picture. We must integrate it with other findings," (Participant 4) suggested a physician. Another participant elaborated, "Using CRP alongside patient history, physical examination, and other diagnostic tests ensures a more accurate and comprehensive assessment" (Participant 10). This balanced perspective was echoed by another physician who noted, "CRP is valuable, but it should complement, not replace, our clinical judgment and other diagnostic tools" (Participant 3). The participants realized that the balance between clinical examinations and laboratory data interpretation should be respected in order to manage patients effectively. These insights highlight the importance of viewing CRP measurements within the context of the overall clinical picture, promoting a more nuanced and practical approach to patient care.

Cultivating a Habit of Questioning Necessity

Critically assessing the need for each CRP test and indeed all tests were seen as crucial for improving clinical decision-making. "Before ordering a test, I now ask myself if it's essential. This habit helps refine my clinical judgment," (Participant 3) noted a participant. Another physician added, "Questioning the necessity of each test encourages us to be more thoughtful and deliberate in our approach, which ultimately leads to better patient care" (Participant 4). This reflective practice was echoed by another participant who remarked, "By constantly evaluating whether a CRP test is warranted, we not only avoid unnecessary procedures but also enhance our diagnostic skills and confidence" (Participant 3). Through clinical experiences in general medicine, general physicians realized that CRP should be used carefully for their clinical advancement, and reconsidering the usage can improve their clinical skills. These insights underscore the importance of questioning the necessity of tests to foster more thoughtful, efficient, and effective clinical practice.

Refining Clinical Reasoning

Reflecting on the necessity and implications of CRP measurements enhanced physicians' clinical reasoning skills, contributing to their overall professional development. "Thinking deeply about why we measure CRP and its relevance to the patient's condition sharpens our clinical acumen," (Participant 11) concluded a participant. Another physician shared, "By carefully considering the context in which we order CRP, we develop a more nuanced understanding of its role in patient care" (Participant 7). This introspective approach was further emphasized by another participant who noted, "Evaluating the significance of CRP results within the broader clinical picture allows us to make more informed and precise decisions" (Participant 9). The participants realized that continual reflection on their clinical reasoning regarding the usage of CRP could deepen their understanding of patient’s medical conditions and enhance their clinical skills. These insights highlight the value of reflection in refining clinical reasoning, promoting a more thoughtful and practical use of diagnostic tools in practice.

## Discussion

Our study aimed to elucidate general physicians' perceptions of CRP measurement in assessing patient conditions, highlighting its diagnostic value, associated dilemmas, and impact on clinical growth. The thematic analysis revealed three main themes: usefulness for diagnosis and collaboration, dilemmas associated with CRP usage, and clinical growth by reconsidering CRP's importance. These findings align with previous literature and provide insights into how general physicians integrate CRP testing into their practice.

The study participants emphasized CRP's role in distinguishing between inflammatory and non-inflammatory diseases, facilitating communication with specialists, and predicting clinical courses. This corroborates findings from previous studies that have established CRP as a valuable diagnostic tool in various clinical scenarios, including infections and inflammatory conditions [16,17]. CRP's utility in enhancing communication with specialists by providing a common understanding of patient severity further underscores its importance in clinical practice [18]. As noted in other studies, this shared understanding is crucial in ensuring timely and effective patient care [18]. In general medicine, negative impressions regarding CRP among general physicians can be caused by the indistinguishable usage of laboratory tests, causing misleading clinical diagnosis and management [19]. As this article shows, effective communication through the use of CRP in simple cases should be promoted, and the clinical conditions should be discussed among medical professionals, including general physicians.

Despite its usefulness, the study highlighted several dilemmas associated with CRP usage. Physicians reported confusion when CRP results did not align with clinical symptoms, leading to potential over-reliance on laboratory results over clinical judgment. This issue has been previously documented, with studies indicating that CRP can sometimes be misleading due to its variability and the influence of non-specific factors [20,21]. The habitual use of CRP driven by training and expectations from senior doctors also contributes to unnecessary testing and resource wastage, a concern echoed in the literature [22]. In the present medical education system, laboratory medicine is taught effectively based on the Choosing Wisely campaign to lower the cost of medicine [10].

On the other hand, on-the-job education may provide different learning opportunities for physicians [23]. Middle to older physicians tend to depend on CRP in clinical decisions, and young physicians may learn the attitude based on apprenticeship and legitimate peripheral participation [24]. This highlights the need for more balanced and judicious use of CRP to prevent over-testing and maintain clinical skills. To improve the usage of CRP and clinical decision-making, continual on-the-job training should be revised to use medical resources effectively, including various medical professionals.

Clinical growth through the reconsideration of CRP's importance is critical to effective general medicine. The study participants recognized the need to view CRP as one of many diagnostic tools, integrating it with patient history, physical examination, and other tests to form a comprehensive assessment. This balanced approach in general medicine is essential for maintaining high standards of care and is supported by previous research emphasizing the importance of holistic patient evaluation [25,26]. Cultivating a habit of questioning the necessity of CRP tests and reflecting on their implications enhances clinical reasoning skills and promotes professional development [27]. This reflective practice is crucial for improving clinical decision-making and is effective in other studies focusing on diagnostic tools in clinical practice [28]. As this article shows, discussion at the bedside among general physicians can improve their learning of assessing clinical courses and the usage of CRP to follow patients' conditions. For the practical usage of CRP, continual reflection and bedside discussion can be driven among general medicine teams [29,30].

Our study has several limitations that should be acknowledged. Firstly, the research was conducted in a single healthcare facility, Unnan City Hospital, which may limit the generalizability of the findings to other settings. The participants' experiences and perceptions may not fully represent those of general physicians in different regions or healthcare systems. Secondly, the study relied on self-reported interview data, which may be subject to recall and social desirability biases. Participants may have presented their practices and perceptions in a more favorable light, potentially skewing the results. Lastly, the thematic analysis was conducted by a relatively small research team, which, despite efforts to mitigate bias through rigorous discussion and reflexivity, may have influenced the interpretation of the data.

## Conclusions

This study provides valuable insights into general physicians' perceptions of CRP measurement in clinical practice. While CRP is recognized as a useful diagnostic tool for distinguishing between inflammatory and non-inflammatory diseases, facilitating communication with specialists, and predicting clinical courses, its usage is not without dilemmas. Discrepancies between CRP levels and clinical symptoms, the influence of various factors, and habitual use driven by training can lead to unnecessary testing and diminished clinical skills. However, viewing CRP as one of many diagnostic tools, questioning its necessity, and reflecting on its use can enhance clinical reasoning and promote professional growth. These findings underscore the importance of a balanced and critical approach to CRP measurement, integrating it within a holistic framework of patient assessment to improve patient care and resource management. Future research should explore similar perceptions in diverse healthcare settings and investigate strategies to optimize the use of CRP in clinical practice.
